# Merging lanes for science

**DOI:** 10.3389/frhs.2022.968175

**Published:** 2022-09-06

**Authors:** Justin Knox, Geoffrey M. Curran

**Affiliations:** ^1^Department of Psychiatry, Columbia University Irving Medical Center, New York, NY, United States; ^2^HIV Center for Clinical and Behavioral Studies, New York State Psychiatric Institute, New York, NY, United States; ^3^Department of Sociomedical Sciences, Mailman School of Public Health, New York, NY, United States; ^4^Department of Pharmacy Practice, University of Arkansas for Medical Sciences, Little Rock, AR, United States; ^5^Department of Psychiatry, University of Arkansas for Medical Sciences, Little Rock, AR, United States

**Keywords:** hybrid implementation-effectiveness designs, COVID-19, vaccines, vaccine hesitancy, implementation science

## Introduction

In a recent editorial published in Science (1), Proctor and Geng argue that COVID-19 has made evident that we need to more formally prioritize implementation research, the study of the uptake of evidence-based interventions, to ensure that our nation's health discoveries are fully realized. Relatedly, Dr. Francis Collins, head of the National Institutes of Health, recently acknowledged that we have underinvested in research on human behavior, as evidenced by the 60 million eligible Americans who have not been vaccinated against COVID-19 despite the widespread availability of safe and effective vaccines. While Proctor and Geng propose a new lane for science, made possible through significant changes to NIH-funding priorities, we argue that methodological approaches within implementation research also need prioritization. To build on their metaphor, in addition to building a new lane for science, we could also merge lanes to improve science.

## Examples

One example of this would be greater use of hybrid effectiveness-implementation designs (2), which blend elements of clinical effectiveness and implementation research to foster more rapid translational science gains. Had COVID-19 vaccine efficacy trials used hybrid designs to assess implementation challenges and efficacy, we could have anticipated some of the implementation barriers that emerged sooner. For example, the trials could have assessed the potential reach of the COVID-19 vaccines, surveying those who refused to participate in the trials, which would have shown that a sizable proportion of Americans would be vaccine-hesitant. We also could have captured covid-specific vaccine hesitancy concerns and created vaccine hesitancy mitigation strategies more specifically and rapidly. Table 1 provides a more detailed description of the implementation data that could have been collected had COVID-19 vaccine efficacy trials used hybrid designs. While we lament this missed opportunity, we note that we could correct this moving forward. For example, we could use hybrid effectiveness-implementation designs more frequently in future vaccines and other clinical trials.

**Table 1:** Examples of data that could be collected as part of a hybrid effectiveness-implementation study on COVID-19 vaccination.

Effectiveness*	Ratio of those assigned to the COVID-19 vaccine arm compared to the placebo arm of who experience: •COVID-19 infection •COVID-19-related hospitalization •COVID-19-related death
Acceptability	• Reasons for refusal (among those who refused) • Patient satisfaction questionnaires regarding the delivery of the COVID-vaccine (among those enrolled)
Feasibility	• Ratio of individuals screened eligible to those enrolled in the trial • Time required to recruit participants
Fidelity	• COVID-19 vaccine delivered as intended, including timing of the second dose (if applicable)
Implementation factors	• Cost of delivering the COVID-19 vaccine (e.g., staffing, training, storage). • Patient need/demand for COVID-19 vaccine • Incentives for providing the COVID-19 vaccine • Compatibility of COVID-19 vaccine with existing workflows and systems • Readiness for implementation of COVID-19 vaccine • Patient knowledge and beliefs about the COVID-19 vaccine • Provider self-efficacy to offer patients COVID-19 vaccine

*Data collected as part of the standard COVID-19 vaccine efficacy trials.

Data could be collected as part of routine study implementation, as well as enhanced by additional primary data collection (e.g., semi-structured interviews with healthcare providers at COVID-19 vaccine trial implementation sites).

## Discussion

Further application of innovative approaches like hybrid effectiveness-implementation designs could supplement the actions called for by Proctor and Geng.

## Author contributions

Conceptualization and writing—original draft: JK. Writing—review and editing: JK and GC. All authors contributed to the article and approved the submitted version.

## Funding

This study was supported by National Institutes of Health grant K01AA028199 (JK) and UL1TR003107 (GC).

## Conflict of interest

The authors declare that the research was conducted in the absence of any commercial or financial relationships that could be construed as a potential conflict of interest.

## Publisher's note

All claims expressed in this article are solely those of the authors and do not necessarily represent those of their affiliated organizations, or those of the publisher, the editors and the reviewers. Any product that may be evaluated in this article, or claim that may be made by its manufacturer, is not guaranteed or endorsed by the publisher.
